# Bridging the Evidence-Practice Gap in Breastfeeding After Anesthesia: A Case Report and Practical Framework for the Non-obstetric Provider

**DOI:** 10.7759/cureus.106789

**Published:** 2026-04-10

**Authors:** Lisa Bethea

**Affiliations:** 1 Anesthesiology, Moffitt Cancer Center, Tampa, USA

**Keywords:** breastfeeding, dexmedetomidine, multimodal anesthesia, perioperative counseling, relative infant dose, sugammadex

## Abstract

Despite updated recommendations from major professional societies, outdated guidance to interrupt breastfeeding after anesthesia persists in clinical practice. This discrepancy reflects an evidence-practice gap, particularly in non-obstetric perioperative settings and in cases involving newer anesthetic agents.

This report describes a 32-year-old lactating female undergoing elective laparoscopic abdominal surgery under general anesthesia using a multimodal approach, including dexmedetomidine and sugammadex. Detailed perioperative management, medication selection, and counseling strategies are presented. Breastfeeding was resumed approximately 60 minutes postoperatively once the patient was awake and neurologically intact, with no observed neonatal adverse effects.

Current recommendations support early resumption of breastfeeding after anesthesia, emphasizing maternal recovery rather than arbitrary time intervals. This case operationalizes these principles in a contemporary anesthetic context and provides a structured framework for perioperative counseling and management. Particular attention is given to agents with limited lactation-specific data, including dexmedetomidine and sugammadex, highlighting the role of pharmacokinetic principles such as relative infant dose and oral bioavailability in guiding clinical decisions.

This case supports existing guideline recommendations and illustrates their application in modern anesthetic practice. It provides a practical, evidence-informed approach to managing lactating patients undergoing non-obstetric surgery while identifying areas where further research is needed.

## Introduction

As breastfeeding rates increase, anesthesiologists are more frequently involved in the care of lactating patients undergoing non-obstetric surgery. Contemporary recommendations from the American Society of Anesthesiologists (ASA) and the Academy of Breastfeeding Medicine (ABM) support early resumption of breastfeeding after anesthesia, emphasizing that interruption is rarely necessary [1,2]. However, outdated advice to “pump and dump” remains prevalent in clinical practice, reflecting a persistent gap between available evidence and perioperative counseling [1,2].

This discrepancy is not due to a lack of pharmacologic data, but rather challenges in translating those data into real-time clinical decision-making. Uncertainty is often amplified in cases involving multimodal anesthetic techniques or agents with less widely recognized lactation data [3,4]. In particular, newer agents such as dexmedetomidine and sugammadex are increasingly used in contemporary anesthetic practice but remain underrepresented in lactation-focused discussions [3,4].

While prior reviews and consensus statements provide general reassurance regarding medication safety, there remains limited literature demonstrating how these principles can be operationalized in routine perioperative care. This case report addresses that gap by presenting a detailed clinical scenario involving modern anesthetic management and by offering a practical, evidence-informed framework for anesthesiologists caring for lactating patients in non-obstetric settings.

## Case presentation

A 32-year-old female presented for elective laparoscopic abdominal tumor resection. Her medical history was notable for a cesarean delivery three months earlier and a laparoscopic appendectomy two weeks prior. She had no chronic medical conditions and was not taking daily medications. She was actively breastfeeding a healthy, full-term infant exclusively, with no history of prematurity or medical comorbidities.

Preoperative evaluation revealed normal vital signs, laboratory values, and airway assessment. During the preoperative interview, the patient expressed concern regarding the safety of anesthetic medications during breastfeeding and whether interruption would be required.

After informed consent was obtained, standard ASA monitors were applied. General anesthesia was induced with intravenous propofol (150 mg), fentanyl (50 mcg total), lidocaine (100 mg), and vecuronium (5 mg), followed by uncomplicated endotracheal intubation. Anesthesia was maintained with sevoflurane in an air-oxygen mixture.

Intraoperatively, the patient received cefazolin (2 gm) and metronidazole (500 mg) for antimicrobial prophylaxis; hydromorphone (2.5 mg total) and ketorolac (30 mg) for analgesia; and famotidine (20 mg) and dexamethasone (4 mg) for postoperative nausea and vomiting prophylaxis. A dexmedetomidine infusion (0.4 mcg/kg/hr) was administered to reduce inhalational anesthetic requirements and enhance analgesia.

The procedure was uneventful, with a total anesthetic duration of approximately five hours. Neuromuscular blockade was reversed with sugammadex (200 mg). The patient was extubated awake and transferred to the post-anesthesia care unit in stable condition.

Approximately 60 minutes postoperatively, the patient was awake, alert, hemodynamically stable, and able to tolerate oral intake. Following targeted counseling based on current ASA and ABM recommendations, she resumed breastfeeding. No neonatal sedation, feeding difficulty, or other adverse effects were observed on follow-up, and breastfeeding continued without interruption.

## Discussion

Guideline-based framework for breastfeeding after anesthesia

Current ASA and ABM recommendations consistently support early resumption of breastfeeding after anesthesia and discourage routine interruption [1,2]. Both organizations discourage routine “pump-and-dump” practices, noting that anesthetic and analgesic agents are transferred into breast milk in clinically insignificant concentrations [1,2,5]. Rather than relying on arbitrary time intervals, these guidelines emphasize that breastfeeding may safely resume once the mother is awake, alert, and able to safely care for her infant [1]. These recommendations emphasize maternal neurologic recovery as the primary determinant of safety rather than drug elimination timelines [1].

Despite this consensus, inconsistent counseling persists, particularly in non-obstetric settings where anesthesiologists may be less familiar with lactation-specific pharmacology [5]. This gap reflects challenges in translating pharmacologic principles into clinical practice, especially when newer or less familiar medications are used [3,6]. The present case illustrates how these guidelines can be operationalized in routine clinical practice, even in the setting of a multimodal anesthetic technique incorporating agents such as dexmedetomidine and sugammadex.

Pharmacokinetic principles and infant exposure

Drug transfer into breast milk is governed by established pharmacokinetic principles, including molecular weight, lipid solubility, protein binding, ionization, and maternal plasma concentration [3,7]. The relative infant dose (RID), defined as the infant’s weight-adjusted dose relative to the maternal dose, provides a practical estimate of exposure. An RID below 10% is generally considered compatible with breastfeeding, although most anesthetic agents fall well below this threshold [3,8]. In addition, many perioperative medications demonstrate poor oral bioavailability, further limiting infant exposure even when small amounts are present in breast milk [3,4]. These principles provide a mechanistic foundation for the safety of breastfeeding after anesthesia.

Application to contemporary anesthetic agents

The medications used in this case are consistent with established safety profiles. Propofol and lidocaine undergo rapid redistribution and clearance, resulting in negligible breast milk concentrations [3,4]. Opioids such as fentanyl and hydromorphone demonstrate low transfer into breast milk following single perioperative doses, although repeated dosing warrants infant monitoring [3,4]. Ketorolac and other nonsteroidal anti-inflammatory drugs are considered compatible and are preferred in multimodal analgesia due to their opioid-sparing effects [7,8]. Volatile anesthetics such as sevoflurane are rapidly eliminated via exhalation and are not expected to accumulate in breast milk [3,4].

The inclusion of dexmedetomidine and sugammadex highlights an area of evolving clinical relevance. Dexmedetomidine appears in low concentrations in breast milk and has poor oral bioavailability; however, direct human lactation data remain limited [4]. Its short context-sensitive half-time and pharmacokinetic profile suggest minimal neonatal exposure in routine perioperative use [4]. Sugammadex, a large hydrophilic molecule with minimal lipid solubility, is expected to have negligible transfer into breast milk and minimal oral absorption by the infant [4]. Nevertheless, current recommendations for both agents rely primarily on pharmacologic inference rather than robust clinical outcome data.

Evidence gaps and clinical implications

This case underscores an important limitation in the current literature: while many anesthetic agents are considered compatible with breastfeeding, this determination is often based on pharmacokinetic modeling rather than direct measurement of infant outcomes [3,4]. As newer agents are increasingly incorporated into multimodal anesthetic regimens, further research is needed to strengthen the evidence base guiding perioperative recommendations.

Perioperative counseling framework

A key implication of this case is the importance of integrating pharmacologic understanding into a practical perioperative counseling framework. Effective perioperative counseling is essential in bridging the evidence-practice gap. Preoperative discussions should address breastfeeding goals, correct misconceptions, and provide reassurance regarding medication safety [6]. Encouraging patients to breastfeed or pump prior to surgery may improve comfort and maintain milk supply.

Intraoperatively, the use of multimodal analgesia can minimize opioid requirements while maintaining adequate pain control, which is itself important for successful lactation [6]. Postoperatively, breastfeeding decisions should be guided by maternal neurologic status rather than predetermined waiting periods [1]. Clear, consistent communication is critical to reducing patient anxiety and supporting informed decision-making [5].

Postoperative prescribing practices further influence infant exposure and should be addressed explicitly. Acetaminophen and nonsteroidal anti-inflammatory drugs are preferred first-line agents due to their safety profiles [8]. When opioids are required, clinicians should prescribe the lowest effective dose for the shortest duration and counsel patients to monitor their infant for signs of sedation or feeding difficulty [2,8]. Even in these circumstances, breastfeeding is generally safe and should not be routinely interrupted. The breastfeeding compatibility of perioperative medications used during general anesthesia is summarized in Table 1.

**Table 1:** Breastfeeding compatibility of perioperative medications used during general anesthesia. This table summarizes the breastfeeding compatibility of commonly used perioperative medications, incorporating relative infant dose (RID), estimated oral bioavailability relevant to infant exposure, and level of supporting evidence. An RID <10% is generally considered compatible with breastfeeding. Most anesthetic agents demonstrate low transfer into breast milk and limited oral bioavailability, supporting early resumption of breastfeeding once the mother is awake and neurologically intact. RID: relative infant dose; IV: intravenous; NSAID: nonsteroidal anti-inflammatory drugs; NMBA: neuromuscular blocking agent.

Medication	Drug Class/Role	Relative infant dose (RID)	Oral bioavailability (Infant)	Breastfeeding recommendation	Level of evidence	Clinical considerations
Propofol	IV induction agent	<0.1% [3,4]	Low [3,4]	Compatible [1-4]	Moderate	Rapid redistribution and clearance; negligible milk levels [3,4]
Fentanyl	Opioid analgesic	<1% [3,4]	Very low [3,4]	Compatible with monitoring [1-4]	Moderate	Short-term use acceptable; monitor infant with repeated dosing [3,4]
Hydromorphone	Opioid analgesic	~2–3% [3,4]	Moderate [3,4]	Compatible with monitoring [1,2,8]	Moderate	Use lowest effective dose; monitor for sedation/apnea [3,8]
Lidocaine	Local anesthetic/IV adjunct	<1% [3,4]	Low [3,4]	Compatible [1-4]	High	Poor oral absorption limits infant exposure [3,4]
Vecuronium	Neuromuscular blocker	Negligible [3,4]	None [3,4]	Compatible [1-4]	Moderate	Highly ionized; minimal transfer into milk [3,4]
Sevoflurane	Volatile anesthetic	Not measurable [3,4]	None [3,4]	Compatible [1-4]	Moderate	Rapid elimination via exhalation [3,4]
Sugammadex	NMBA reversal agent	Low (limited data) [4]	Very low [4]	Compatible [1,2,4]	Limited	Large, hydrophilic molecule; minimal transfer expected [4]
Dexmedetomidine	α2-agonist sedative	Low (limited data) [4]	Low [4]	Compatible [1,2,4]	Limited	Low milk levels; limited human lactation data [4]
Ketorolac	NSAID analgesic	<1% [7,8]	Moderate [7,8]	Compatible [1,2,8]	High	Opioid-sparing; preferred analgesic option [7,8]
Dexamethasone	Antiemetic/anti-inflammatory	Low [3,4]	Moderate [3,4]	Compatible [1-4]	Moderate	Single perioperative doses are considered safe [3,4]
Famotidine	H2 receptor antagonist	<2% [3,4]	Moderate [3,4]	Compatible [1-4]	High	Widely used; minimal infant effects reported [3,4]
Cefazolin	Antibiotic (β-lactam)	<1% [7,8]	Low-moderate [7,8]	Compatible [1,2,7,8]	High	Common perioperative use; safe in lactation [7,8]
Metronidazole	Antibiotic	<10% (single dose) [7,8]	High [7,8]	Compatible [1,2,7,8]	Moderate	Routine perioperative dosing is acceptable [7,8]

## Conclusions

This case supports current ASA and ABM recommendations that breastfeeding may safely resume after anesthesia once the mother is awake and neurologically intact. It illustrates the application of these principles within a modern multimodal anesthetic framework, including agents with limited lactation-specific data.

As a single case report, these findings are illustrative rather than definitive. However, they provide a practical example of evidence-based perioperative management and highlight the importance of translating pharmacologic knowledge into consistent clinical practice. Addressing this evidence-practice gap has meaningful implications for patient experience, maternal-infant bonding, and the preservation of breastfeeding.
